# Epidemiological Surveillance System on Foodborne Diseases in Brazil after 10-Years of Its Implementation: Completeness Evaluation

**DOI:** 10.3390/ijerph15102284

**Published:** 2018-10-17

**Authors:** Cainara Lins Draeger, Rita de Cassia Coelho de Almeida Akutsu, Wilma Maria Coelho Araújo, Izabel Cristina Rodrigues da Silva, Raquel Braz Assunção Botelho, Renata Puppin Zandonadi

**Affiliations:** Department of Nutrition, Faculty of Health Sciences, University of Brasilia, Brasilia 70910-900, Brazil; cainara@gmail.com (C.L.D.); rita.akutsu@gmail.com (R.d.C.C.d.A.A.); wilma.araujo@terra.com.br (W.M.C.A.); belbiomedica@gmail.com (I.C.R.d.S.); raquelbabotelho@gmail.com (R.B.A.B.)

**Keywords:** completeness, epidemiological surveillance system, foodborne disease, health

## Abstract

This study aimed to evaluate the data quality of the Brazilian Epidemiological Surveillance System on Foodborne Diseases (VE-DTA) through the evaluation of the completeness of the record after 10-years of its implementation. The study evaluated the measurement of completeness by quantifying ignored, incomplete or blank responses of the data items filled. The evaluation used the percentage of completion of these items regarding the total number of notifications registered in the system. We organized the results according to the general Category of completeness of the database, by year of notification and region of occurrence. We also evaluated the overall completeness percentages of the database and the completeness levels according to the degree of recommendation of completion of each variable (mandatory, essential, and complementary) by the VE-DTA manual. The system presented 7037 outbreaks of foodborne diseases. According to the completeness classification, the database presented general classification as Category 1 since it has 82.1% (*n* = 5.777) of variables with the level of completion up to 75.1%. We observed that 8.6% of the database was classified as category 2; 9.2% as category 3 and 0.1% as category 4. The improvement on database quality regarding completeness can positively impact on public health and public policies, reducing the number of FBDs deaths.

## 1. Introduction

When one or more consumers experience similar symptoms after the ingestion of contaminated food or water, it is characterized as a Foodborne Diseases (FBD) [1]. Epidemiological data on FBD is often minimally collected in developing countries. Even the most obvious foodborne outbreaks usually are not adequately investigated, not reported or go undetected [2]. Enhancing FBD surveillance and improving the timeliness of outbreak detection have been identified as public health priorities [3].

Due to the increasing number of cases of FBDs in Brazil, the Health Surveillance Secretariat of the Department of Health developed in 2007 the National Epidemiological Surveillance System for Foodborne Diseases (Sistema Nacional de Vigilância Epidemiológica das Doenças Transmitidas por Alimentos—VE-DTA). This system aims to provide information that promotes health actions to reduce the occurrence of FBDs in the country [4]. VE-DTA information is Nationwide, and it is available on the internet for public download (http://portalms.saude.gov.br/saude-de-a-z/doencas-transmitidas-por-alimentos/situacao-epidemiologica). If people want more specific data, they should access another link (https://esic.cgu.gov.br/sistema/site/index.aspx) and fill in the form asking for specific data. The Municipal Secretariat must notify the State Health Secretariat about the foodborne disease outbreaks. It is mandatory for local doctors and health professionals to inform the Municipal State Secretariat about these health events. The health professional must register notified and investigated foodborne outbreaks; place of occurrence; the number of affected people (gender and age range); the number of hospitalized people; the number of deaths; the primary clinical manifestations; the etiologic agents and the food involved [5].

After the registration in the VE-DTA system, the Municipal Health Secretariats must send weekly the files with the registered information to the State Health Secretariats. Communication between the State Health Secretariats and the Federal control occurs every two weeks (Figure S1). If no cases of illness occur, health units must complete a negative notification form. This is a strategy designed to show that the health professionals are alert to the occurrence of such events and to avoid underreporting. Moreover, if the municipalities do not fill in the database for two consecutive months, the government suspends the financial resources for the operationalization of health units, according to Brazilian legislation [5].

Databases from health information systems are low-cost technological tools that allow a high volume of information about the population’s health situation. These tools are essential since they can be used for planning, organizing, operating, and evaluating governmental actions, services, programs, and policies. However, for the effective use of the information, it is essential to ensure that the data are valid and reliable. Data quality is necessary for an accurate assessment of the status of the nation [6].

Surveillance systems should be efficient, flexible, and scalable. However, many current systems are slow and inefficient. They use out-of-date technologies that no longer meet user’s needs for data management, analysis, visualization, and dissemination [7]. Data management and analysis must be designed and executed with extreme care as errors could have a devastating impact on public health or the food industry. Therefore it is important to establish quality parameters that clearly define the quality of a database [8].

Completeness evaluation of health information systems is one of the attributes of database quality evaluation, and it should be examined by the quantification and quality of the completion of the items (Example: the proportion of notified cases without fulfilling the criterion of confirmation) [9].

A good quality database must be complete (contain all cases diagnosed); reliable to the original data recorded in the health units (reliability); without duplicities; its items must be complete and consistent. It is essential to analyze the quality of the database to identify and to solve data inconsistencies and duplication of the data records [5].

To the best of our knowledge, no research has evaluated the completeness of the epidemiological surveillance system of foodborne diseases in Brazil. Due to the absence of this kind of study in Brazil and the importance that these data have for the population, the present study aims to evaluate the data quality of the VE-DTA system in Brazil by the evaluation of its records completeness after 10-years of its implementation.

## 2. Materials and Methods

A cross-sectional study was performed analyzing data from the Brazilian Epidemiological Surveillance System on Foodborne Diseases (VE-DTA), from its inception until December 2016.

The quality of all FBD data (from January 2007 to December 2016) was analyzed using the information from the VE-DTA platform downloaded on January 2018 [4]. The evaluated variables are part of a standardized form with (i) mandatory items; the absence of these items do not allow registration of the notifications in the system database; (ii) essential items; they are optional and show important data to the VE-DTA investigation and the calculation of epidemiological indicators; (iii) and complementary items; they are not mandatory neither essential, but they are included in system to help understanding the VE-DTA. These items mandatory, essential and complimentary can complement information about each individual cases. The number of variables corresponding to each item, its level of recommendation of completion and the summary of the information required by a group of variables are in Table 1. It is important to highlight that the variables presented in each category are the main variables of the database. However, the database presents secondary variables derived from the main variables to compose the total number of variables.

According to the Guidelines for Evaluating Public Health Surveillance Systems created by the Center for Disease Control and Prevention (CDC), high-quality data comes from high response rates. Therefore, completeness is crucial for data quality. The best category from the Brazilian VE-DTA System is category 1 followed by 2, 3, and 4 [4,5,7,10].

**Figure 1:**
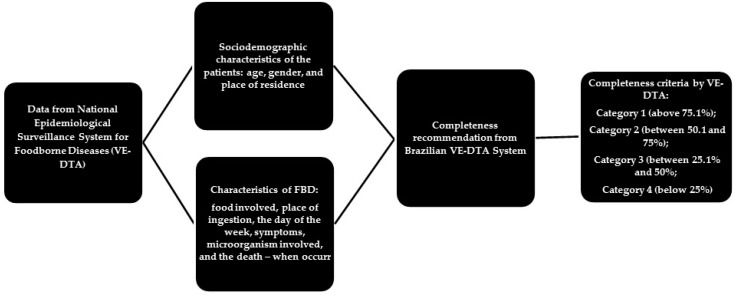
Stages of the data evaluation process from the Brazilian Epidemiological Surveillance System on Foodborne Diseases (VE-DTA).

We measured completeness by quantifying ignored, incomplete or blank responses in the items [11]. According to the classification suggested by VE-DTA platform [12], the evaluation was based on the percentage of completion of the items regarding the total number of notifications registered in the system, considering the following filling classification criteria: Category 1 (above 75.1% completion); Category 2 (between 50.1 and 75%); Category 3 (between 25.1% and 50% and Category 4 (below 25%) [13]. Figure 1 shows the stages of the data evaluation process from the Brazilian Epidemiological Surveillance System on Foodborne Diseases (VE-DTA). For this evaluation, we considered the items filling with the information “ignored”, coded by numbers 9, 99, or empty, non-complete [11]. We used SPSS^®^ software version 22.0 (IBM, Armonk, NY, USA) for data processing and analysis.

## 3. Results

The present study provides the first quality data assessment from the Brazilian VE-DTA. In the 10-year period (from January 2007 to December 2016), 7037 outbreaks of foodborne diseases were reported in the system. From the notification of the outbreak, the sanitary and epidemiological surveillance begins with the local investigation of the place where the suspicious food was produced to gather possible hygienic-sanitary conditions, sample collection (when there are leftovers), interview with the people involved, clinical and laboratory data collection (if complementary tests were performed), constituting 108 investigation variables to be filled in the system (VE-DTA). We measured completeness with these data. We organized the results according to the general Category of completeness of the database, by year of notification and region of occurrence of the outbreak. We also evaluated the overall completeness percentages of the database and the completeness levels according to the degree of recommendation of completion of each variable (mandatory, essential, and complementary) by the VE-DTA manual. According to the completeness classification, the database presented general classification as Category 1 since it has 82% (*n* = 5.777) of variables with the level of completion. We observed that 8.6% of the database were classified as category 2; 9.2% as category 3 and 0.1% as category 4.

**Figure 2:**
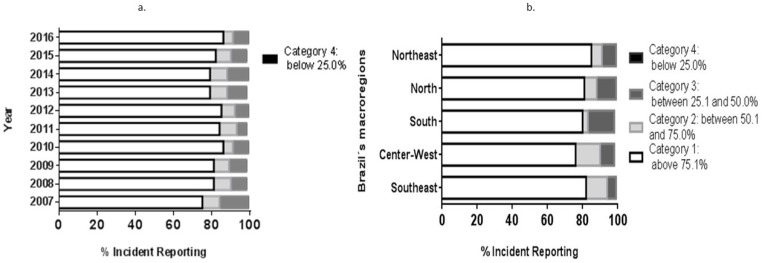
National Epidemiological Surveillance System for Foodborne Diseases (VE-DTA) completeness classification by notification year (**a**) and Brazilian regions (**b**), Brazil 2007–2016. Note: Category 4 is below 0.5%.

There is a significant difference between the years 2007 to 2016 (*x*^2^ = 98.058; *gl* = 27; *p* = 0.000) regarding completeness by year of the notification (Figure 2a). Proportionally, the year that had the highest occurrence of Category 1 classification was 2016, with 87% of completeness; and the year with the lowest completion of Category 1 was 2007 with 75%.

**Figure 3:**
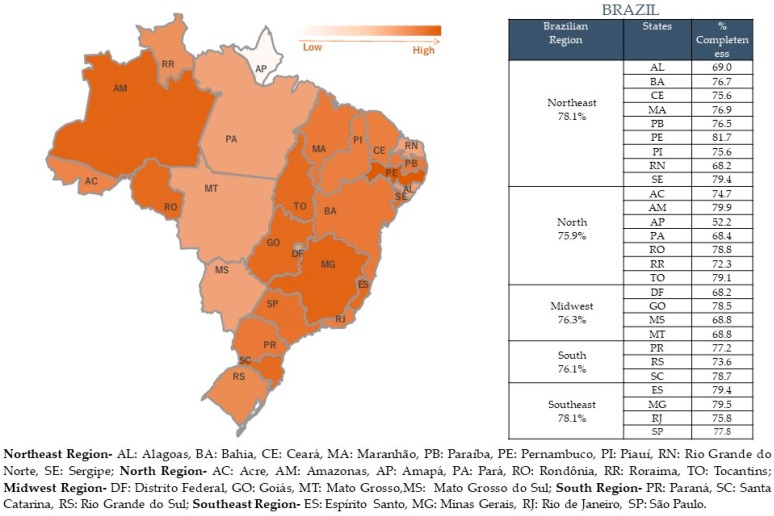
Total completeness % of all the States by region.

There is also a significant difference between the Brazilian regions (*x*^2^ = 278,204; *gl* = 12; *p* = 0.000) (Figure 2b). The Northeast region had the highest occurrence of category 1 completeness notifications (85% of notifications), even though the total completeness notification for this region is 78%, as well as for the Southeast region (Figure 3). The Midwest had the worst result for Category 1 completeness. In general, all the observed regions had a high percentage of total completeness data (Figure 3).

**Figure 4:**
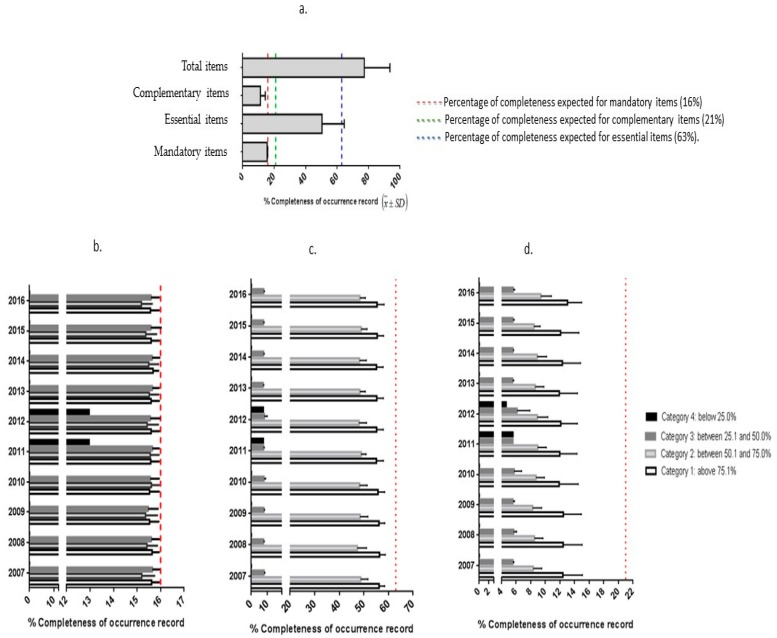
Percentage of database completeness according to the degree of recommendation of completing the variables (complementary, essential, and mandatory (**a**)). Percentage of the mandatory (**b**), essential (**c**) and complementary (**d**) variables classified in Category 1, 2, 3 and 4 of completeness on National Epidemiological Surveillance System for Foodborne Diseases (VE-DTA).

The analysis of the overall completeness database showed that 77% (mean *n* = 83.51; standard deviation = 17.61) of data were completed from the 108 variables, (Figure 4). When evaluating the completeness of the variables by the level of recommendation, we observed that variables of mandatory completion had greater completeness than variables of essential or complementary filling.

Figure 4a shows the percentage of completion of each group of variables. We emphasize that the essential items had the lowest completeness when compared with the expected percentage (blue line, Figure 4a). Mandatory items presented almost the total of the expected completeness (red line, Figure 4a), since they are required by the VE-DTA. Figure 4b–d demonstrates the classification results in the completeness of the variables (Categories 1 to 4) according to the three levels of recommendation for completing variables (mandatory, essential, and complimentary) from the VE-DTA manual over the years 2007–2016. In general, for the required items, the results are close to the expected (100% completeness for the mandatory items).

In Figure 4c, d, there is a statistical difference between the average filling of the mandatory items according to the category and the year (*p* < 0.0001) and the essential items according to category and year (*p* = 0.014). In Figure 4d, there is a statistical difference in the mean of completeness of the complementary items according to the category within the year, but not over the years, since the standard is maintained (*p* = 0.2692).

The variables with the highest percentage of non-completion are: the specification of other sites of outbreaks occurrence, with 100% incompleteness; the number of positive samples of microbiological analyzes with rates above 96% of incompleteness, and specification of the etiological agent with rates above 98% of incompleteness.

The best data completeness rates, with 100% completeness, are related to the primary notification registry variables, such as: identification of the grievance; date of notification; type of notification; epidemic week of the notification; year; Federation Unit; date of first symptoms; opportunity for notification; number of suspected cases exposed by the date of notification; date of the investigation; research opportunity; mode of transmission; vehicle of the transmission mode when indirectly; results of clinical findings; result of the first analysis of the etiological agent causing the outbreak; and an opportunity for closure of the case.

## 4. Discussion

The variables with the best classification for completeness were mostly variables of individual identification of the patients, which is mandatory information defined in the manual system. This fact may justify its high level of completeness [4]. Despite that, for the classification on category 1 of VE-DTA completeness, we considered that a general completeness level of 77% is unsatisfactory since it is a database with records of health problems with a high level of morbidity in the Brazilian population [14]. To correctly and completely evaluate an outbreak, we need all the information of the system. As an example, even though completeness of category 1 was high, the microbiological analysis was not complete in the system. How can we prevent or treat people if we do not know the microorganisms of the outbreak?

The foodborne diseases present symptoms that may range from mild gastroenteritis to more severe situations such as the development of renal, hepatic, neurological damage, and death. They represent one of the most common and significant public health problems in the world, especially in developing countries that still have severe shortcomings in infrastructure and basic sanitation. In general, the countries that present the highest occurrence rates are the ones that have the least resources to prevent them [12,15]. These data highlight the magnitude of the problem and the importance of improving the classification of completeness for adequate monitoring of FBDs.

Several factors such as environmental changes, industrialization, habit changes, lifestyle, behaviors of food handlers and also lack of information for the population on the risks characterize the incidence of FBD [16]. Inadequate registration of cases makes it difficult to adopt adequate measures to correct the problem, negatively impacting on public health.

We did not find other studies evaluating the completeness of FBD data worldwide. However, comparing the completeness data from the present study with the completeness of other studies that assessed the completeness data in other health conditions [17,18,19], it is noteworthy that the completeness of the FBD still needs to be improved and studied.

Depending on the type of health problem, the completeness of data is directly affected. A review study on data completeness published by the US CDC in 2014 highlighted that health conditions related to AIDS, tuberculosis, and varicella were higher than other diseases. It can be justified by the existence of well-established disease control programs with wide-national dissemination. An example of this is the need to publicize the monitoring program for FBDs since they are diseases with high morbidity and under-reported in the systems [20].

Regarding the FBD notifications by the Brazilian regions, we observed that the Northeast region obtained the highest levels of completeness of the variables and the Southeast region presented the lowest number of FBD notifications. That occurs due to the presence of the highest number of FBD notifications in the Southeast region. It is important to highlight that the low quality of data increases the number of errors in the decision-making process, reducing the attention level of the population health care [21]. The high percentage of the incompleteness of microbiological analyzes (above 96%), and specification of the etiological agent (rates above 98% of incompleteness) indicates that either no laboratory testing is being done or that the results are not being reported. A foodborne disease outbreak database without any information on testing or etiological agent is not very helpful to guide public policies.

Therefore, monitoring systems are considered important sources of secondary data for health analysis and research. In addition to the broad population coverage, the system information is easily accessible and obtainable. However, it is the evaluation of the quality of the available data that is very important to avoid that they are incomplete or inconsistent. If the quality of the information is not considered, the obtained results may not represent the reality, thus compromising the knowledge and health actions [22].

The analysis shows that general completeness did not improve over time. The government expected an improvement in the completeness once the system began in 2007 and over time health professionals were already better adapted and aware of the importance of using the system [4]. Unfortunately, there was no overall improvement of completeness. We observed that the group of variables that were the most complete was those of mandatory completion, while the others were incomplete (essential and complementary). This highlights that it is necessary to make the other variables also mandatory variables since they are also fundamental for a complete epidemiological investigation of the outbreaks of DTA.

Those responsible for completing the VE-DTA System are workers of the municipal and regional health secretariats, and they have a medium to high level of education. Detailed information about the profile of the users as well as the exact level of schooling is not available for consultation, constituting a limitation for the analysis of the degree of instruction and a possible association with the completeness of the VE-DTA System. According to the CDC guidelines for evaluating public health systems, the quality of training and supervision of the people who complete these surveillance forms influence the quality of data. Therefore, a review of these aspects, in a public health surveillance system, could provide an indirect measurement of data quality. Additional staff training providing better instructions as well as explaining the importance of data base completeness to the management of future outbreaks would probably improve compliance with regulation and the quality of the VE-DTA [11]. In addition, systematic monitoring of database and real-time feedback to data submitters would also likely improve data quality. Completeness monitoring is a valuable tool to verify if data is being adequately completed and identifying systems failures, expanding its use and improving strategies in the generation and dissemination of information [6].

## 5. Conclusions

This is the first study that evaluated the completeness of the National Epidemiological Surveillance System for Foodborne Diseases (VE-DTA). Studies on the completeness of the data contribute to the identification of failures on the data collection process due to the lack of knowledge about the filling, changes in the pattern of disease notification, and ability to operate the database. Although 7037 outbreaks of foodborne diseases are in the system, most of them (77.32%) were not adequately completed. The highest levels of completeness were for the mandatory variables. These data highlight the need to improve the system completeness quality and the obligation of adequate data registration for FBDs monitoring. The system had a high cost for the government, as well as a high cost for training on how to use it. Therefore, incomplete and incorrect fillings do not improve decision making by the government to improve health conditions. It is necessary not only to develop systems but also to evaluate their use to have good materials for decision making. The improvement on database quality regarding completeness can positively impact on public health and public policies, reducing the number of FBDs deaths.

## Figures and Tables

**Table 1:** Summary of information on mandatory, essential, and complementary items of the VE-DTA.

Mandatory Items	Essential Items	Complementary Items
Summary of data to be completed: Notification number; Name of the grievance or illness being notified; Date of notification; Place of occurrence of the outbreak; Municipality of notification; Notification health unit; Date of first symptoms; Municipality of occurrence of the outbreak; Country of residence of the patient; Research start date; Research opportunity.	Summary of data to be completed: Number of patients, hospitalized patients, deaths, patients according to age range and gender; Signs and symptoms presented by patients; Observed incubation period; Place of production or preparation of suspect foods; If clinical samples were collected (yes, no, ignored); Main finding in clinical samples and other findings; If food samples were collected (yes, no, ignored); Main finding in food samples; Etiologic agent of the outbreak; Food causing the outbreak; Criterion of confirmation of the etiological agent; Date of closure of the outbreak.	Summary of data to be completed: Code corresponding to the type of notification; Total suspected cases up to notification; Place of occurrence of the outbreak and details of where the outbreak occurred: District, neighborhood, public place, street number, geo-referencing field, reference point, zip code, telephone, zone (urban, rural or periphery); Mode of disease transmission: direct, indirect and ignored; Whether indirect transmission, transmission vehicle; Total number of people and patients interviewed; Longest incubation period observed in a patient; Place of ingestion of suspect foods; Probable causal factors for contamination of suspect foods; Number of clinical and food samples collected and number of positive findings; Measures adopted or recommended in the Outbreak; Name of the health unit responsible for the investigation; Code of the health unit responsible for the investigation; Full name, function and signature of the person in charge of the investigation.
Total: 17 variables (16% of total items)	Total: 68 variables (63% of total items)	Total: 23 variables (21% of total items)

## Supplementary Materials

The following are available online at http://www.mdpi.com/1660-4601/15/10/2284/s1, Figure S1: Registration and communication flow of foodborne diseases in the Brazilian VE-DTA system.
