# Supplementary material for: Epidemiological Surveillance System on Foodborne Diseases in Brazil after 10-Years of Its Implementation: Completeness Evaluation
Source: Int J Environ Res Public Health. 2018 Oct 17;15(10):2284. doi: 10.3390/ijerph15102284 (PMC6210259; doi:10.3390/ijerph15102284)
Supplement: Supplementary file 1 [file ijerph-15-02284-s001.pdf]

Supplementary

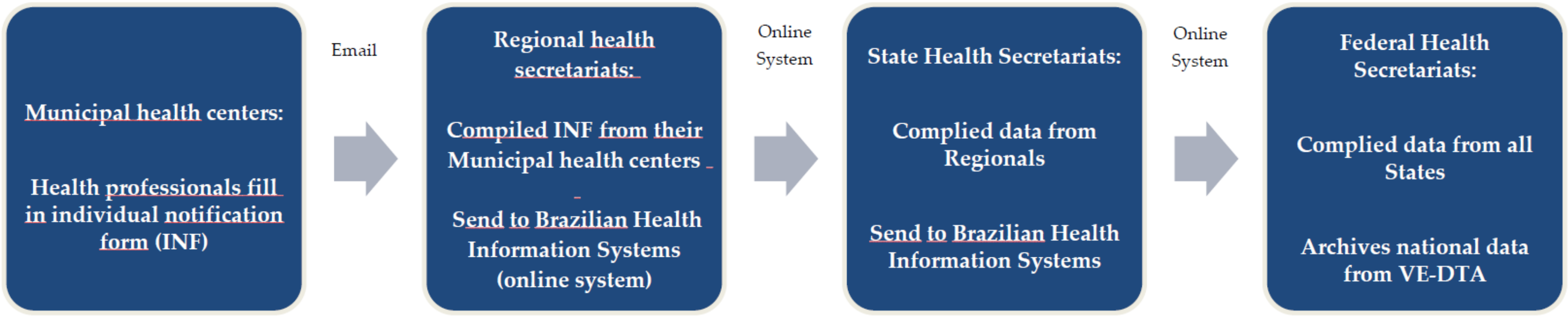

Figure S1. Registration and communication flow of foodborne diseases in the Brazilian VE-DTA system.
